# Common mechanisms underlying diabetic vascular complications: focus on the interaction of metabolic disorders, immuno-inflammation, and endothelial dysfunction

**DOI:** 10.1186/s12964-022-01016-w

**Published:** 2023-10-30

**Authors:** Chongxiang Xue, Keyu Chen, Zezheng Gao, Tingting Bao, LiShuo Dong, Linhua Zhao, Xiaolin Tong, Xiuyang Li

**Affiliations:** 1 Institute of Metabolic Diseases, Guang’anmen Hospital, China Academy of Chinese Medical Sciences, No.5 BeiXianGe Street, Xicheng District, Beijing, 100053 China; 2 Beijing University of Chinese Medicine, Beijing, 100029 China; 3 Department of Endocrinology, Guang’anmen Hospital, China Academy of Chinese Medical Sciences, Beijing, 100053 China; 4 Changchun University of Traditional Chinese Medicine, Changchun, 130117 China

**Keywords:** Diabetes mellitus, Diabetic vascular complications, Common mechanisms, Endothelial dysfunction, Secondary prevention

## Abstract

Diabetic vascular complications (DVCs), including macro- and micro- angiopathy, account for a high percentage of mortality in patients with diabetes mellitus (DM). Endothelial dysfunction is the initial and role step for the pathogenesis of DVCs. Hyperglycemia and lipid metabolism disorders contribute to endothelial dysfunction via direct injury of metabolism products, crosstalk between immunity and inflammation, as well as related interaction network. Although physiological and phenotypic differences support their specified changes in different targeted organs, there are still several common mechanisms underlying DVCs. Also, inhibitors of these common mechanisms may decrease the incidence of DVCs effectively. Thus, this review may provide new insights into the possible measures for the secondary prevention of DM. And we discussed the current limitations of those present preventive measures in DVCs research.

Video Abstract

**Supplementary Information:**

The online version contains supplementary material available at 10.1186/s12964-022-01016-w.

## Introduction

With the enhancement of our lifestyle, the global epidemic of metabolic diseases as represented by DM and its complications seriously threatens human life and health [1]. According to International Diabetes Federation (IDF 2021, version 10th), it is estimated that 537 million of the adult population were affected [2]. The high incidence of DM imposes huge personal and societal healthcare burdens. Global health spending on adult DM has grown from US$ 232 billion in 2007 to US$ 966 billion in 2021, representing a 316% increase over 15 years [2]. As for World Health Organization (WHO) statistics from 2005 to 2015, the economic burden of DM on our country was 557.7 billion US dollars, of which 80% was spent on diabetic complications [3].

DVCs, including microangiopathy (diabetic kidney disease (DKD), diabetic retinopathy (DR), diabetic cardiomyopathy (DCM), etc.) and macroangiopathy (DM-related coronary heart disease (CHD), etc.), play a leading role in morbidity and death among patients with DM [4–6]. Adults (20–79 years old) who died from DM or its complications equate to 12.2% of all-cause deaths in 2021 [2].

In recent years, the three-level system for prevention and control of DM has been proposed, in which secondary prevention (prevention of complications) and tertiary prevention (treatment of complications, delaying their death and disability) are both focused on the stage of complications [7]. Therefore, the early prevention and treatment of diabetic complications, especially DVCs, occupy a concernful position. At present, the intervention of diabetic microangiopathy is mostly at the level of tertiary prevention, such as ACEI/ARB drugs and SGLT2 inhibitors for DKD [8], calcium dobesilate combined with laser photocoagulation and anti-vascular endothelial growth factor (VEGF) agent for DR [9, 10], anti-heart failure therapy for DCM [11]. However, no clear evidence-based proof confirms the preventive ability of these therapeutic measures. Only general practical guidelines that control blood glucose, blood pressure, and blood lipids are recommended for DM secondary prevention, while more effective targeted measures are absent.

Known as one of basic pathological features of DVCs, vascular endothelial dysfunction is ordinarily caused by common upstream mechanisms of metabolic disorders and immuno-inflammation [12–14]. In this article, we reviewed the interaction of metabolic disorders, immuno-inflammation and endothelial dysfunction. By summarizing the common core mechanisms underlying DVCs, we hope to grasp opportunities to find multi-targeted approaches for the prevention and treatment of DVCs.

## Endothelial dysfunction and DVCs

Endothelial cells (ECs) constitute a single-layer cell barrier covering the surface of the vascular lumen [14]. They bear the brunt of various stimuli from DVCs, highlighting their importance on vascular morphology, function, and metabolism homeostasis regulation [15].

There are two important hallmarks closely coordinated with each other to provoke endothelial dysfunction in DVCs: one is hyperglycemia induced by insufficient insulin secretion or insulin resistance (IR), and another is chronic, low-grade inflammation induced by proinflammatory immune cells and released cytokine [16]. It is characterized by reduced production or bioavailability of nitric oxide (NO), increased oxidative stress, increased vascular endothelial growth factor (VEGF), vasomotor dysfunction, and impaired endothelial recovery [17–19].

Macrovascular disease depends on vascular smooth muscle cells (VSMCs) to regulate vasomotor response while tiny blood vessels and capillaries are less affected by vascular tension for a lack of VSMCs [17]. So vascular endothelial homeostasis is more particularly significant to diabetic microangiopathy. For microvessels, the resistance to systemic blood flow in the body's blood vessels increased with high blood pressure. Small artery remodeling results in a decrease in blood flow reserve function, leading to abnormal tissue perfusion, and even preclinical or clinical cardiovascular diseases [17, 20]. Endothelial barriers (including blood–brain, blood-retina, and glomerular barrier) are destroyed for cellular connexin downregulation and integrin upregulation, thereby leading to vascular leakage and abnormal permeability [21, 22]. For diabetic macroangiopathy, endothelial dysfunction leads to vascular wall remodelling. Subsequently vascular becomes stiffer and less compliant, and even atherosclerosis (AS) forms [20, 23]. Thus, ECs are potential therapeutic targets for the prevention and treatment of DVCs, whether they are macrovascular or microvascular diseases.

## Regulatory mechanisms of vascular endothelial homeostasis in DVCs

**Fig. 1:**
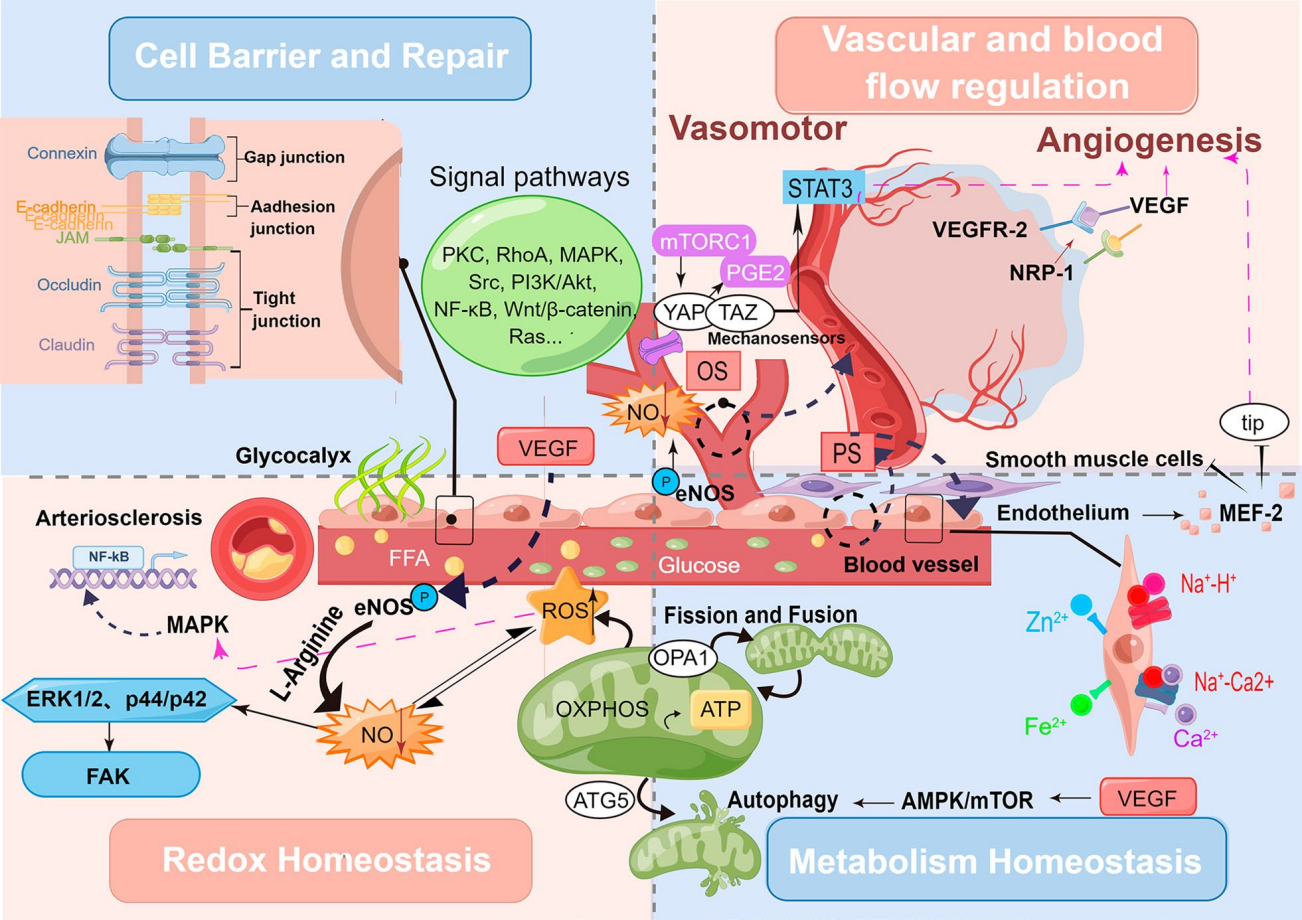
Four main regulatory mechanisms of vascular endothelial homeostasis. Mechanisms required for endothelial homeostasis regulation include cell barrier and repair homeostasis, vascular and blood flow regulation homeostasis, redox homeostasis and metabolism homeostasis; cell barrier and repair involves cell-to-cell junction, communications and relative pathways; vascular and blood flow regulation homeostasis involves blood flow sensor and control, as well as angiogenesis; redox homeostasis involves intracellular and extracellular reduction–oxidation reaction associated with inflammation; metabolism homeostasis involves mitochondria balance, energy conversion, metabolite and ion exchange. eNOS: endothelial nitric oxide synthase; FFA: free fatty acid; NO: nitric oxide; NOX: NADPH oxidase; NRP1: neuropilin-1; OS: oscillatory shear; PGE2: prostaglandin E2; PKC: protein kinase C; PS: Pulsatile shear; ROS: reactive oxygen species; YAP: Yes-Associated Protein; VEGF: vascular endothelial growth factor; VEGFR2: VEGF receptor 2

The maintenance of vascular endothelial homeostasis in DVCs, a complex regulatory process involving multiple steps, includes cell barrier and damage repair, vascular and blood flow regulation, redox, metabolic regulation and so on (Fig. 1).

### Cell barrier and damage repair homeostasis

The normal cell barrier ensures complete structure and well function of vascular endothelium, which is the basis of vascular endothelial homeostasis [24]. Inner cell-to-cell connections include tight junction (TJ), adherens junction (AJ), and gap junction (GJ) [25]. Normally arranged ECs rely on tight junctions to maintain paracellular permeability and polarity, forming an inner membrane barrier [25]. TJ regulation-related signal pathways involved in DVCs include the protein kinase C (PKC), RhoA, MAPK, Src, PI3k/Akt, NF-κB and so on [24, 26]. AJ controls cell contact inhibition and permeability of inflammatory cytokine and solutes. Uncontrolled DM and DVCs cause inactivation and reduction in the expression of TJ protein, and related regulative signal pathways include Wnt/β-Catenin, PI3k/Akt, Ras, Rac, RhoA and so on [25, 27]. The endothelial glycocalyx (EG), which is associated with GJ, acts as a special barrier with glycosaminoglycans attached to prevent DVCs [28]. EG maintains structural and functional integrity of gap connexin proteins, ensuring interendothelial molecular transport [29]. More importantly, EG can reduce platelet and leukocyte adhesion, inflammatory stimulation, and oxidative stress damage to vascular ECs, underscoring the importance of EG preservation to avoid disease initiation and progression [30, 31].

Apart from cell-to-cell junctions, maintaining barrier function needs updates and repairs of vascular ECs themselves. The sphingolipid signal pathway and related mediators, such as sphingosine 1-phosphate (S1P) and its G-protein coupled receptor 1 (S1PR1), perform barrier protective function to ECs and restore cell communication [29, 32]. Activation of S1PR1 prompts redistribution of connexins (VE-cadherin, β- Catenin, and α-Catenin, etc.) to the intercellular contact region, thereby tightening ECs barrier [33]. Concerning ECs repair, activation of transcription factors EGR1 and STAT3 is needed for transcribing sphingosine kinase 1 and sphingolipid transporter 2 to increase the generation and efflux of S1P [34, 35]. Then, the transition of S1PR1^+^ ECs amplifies and they activate endothelial regenerative programs to mediate vascular endothelial repair in DM [35, 36]. In addition, the role of immune cells and circulating angiogenic cells (CACs) are even more crucial to the sustainable complement for ECs [37, 38]. As one of the necessary regulatory factors maintaining the chemotaxis of CACs, endothelial nitric oxide synthase (eNOS) activates MMP-2 and MMP-9 to promote bone marrow differentiation and release CACs [30, 32, 38]. CACs position the site of endothelial injury and produce cytokines (VEGF, SDF-1, etc.) to promote angiogenesis and ECs repair in DM [38, 39].

### Vascular and blood flow regulation homeostasis

Mechanical stress is a key factor underlying the pathophysiology of DVCs. Compared to straight vascular segments exposed to laminar flow, other vascular sites exposed to disturbed flow patterns appear more susceptible to endothelial dysfunction and atherosclerotic plaque formation [40]. Pulsatile shear (PS) stress in straight segments increases the expression of antioxidant and anti-inflammatory (flow-mediated genes (e.g. YAP-TAZ)) genes to keep a vascular protective phenotype, whereas this is not the case for oscillatory shear (OS) stress at the vessel curvatures, branchpoints, and bifurcations [41]. The signal transition mediated by various endothelial mechanosensors (e.g., caveolae and lipid rafts, membrane proteins, primary cilia, glycocalyx, cellular junction, and adhesion molecules) is responsible to regulate vascular homeostasis and adaptative ability [42]. Take the YAP protein (one mechanosensor) as an example, it interacts with STAT3 to regulate angiogenesis [43]. YAP/TAZ pathway, constitutively activated by hyperglycemia, has been proven to be associated with proinflammatory gene expression in endothelial cells as well as disturbed flow-induced diabetic vascular inflammation and renal damage [44, 45].

Under acute stress conditions (e.g., trauma, infection, fever), a sensitive mechanotransduction sensing mechanism regulates blood flow changes to match tissue supply–demand balance through negative feedback [46]. Meanwhile, ECs are regulated by sympathetic nerves to synthesize, release and balance endogenous regulators (e.g., NO, PGs, ET-1, and endothelium-derived hyperpolarizing factors) of vascular tone change [47, 48]. Vascular ECs can also secrete platelet-activating factors, and regulate factors related to the coagulation-fibrinolytic system to control blood flow [49].

Under long-term chronic injury, ECs extracellular matrix turnover interacts with smooth muscle cells that control intimal proliferation, thereby affecting vascular structure and function [46]. Numerous regulated signal pathways and targeted molecules (VEGF, FOXO1, and several miRNAs) get involved [50–53]. VEGF, a key factor in angiogenesis and normal vascular function maintenance, binds to its main receptor VEGFR-2 to regulate ECs proliferation and migration [54]. Neuropilin-1 (NRP-1) acts as a co-receptor to increase their binding ability [55]. Another regulatory factor FOXO1 inhibits the MYC signal pathway to decrease over-proliferated ECs and overgrown neovascular, maintaining a quiescent phase [56]. A variety of miRNAs, including miR-126, help to leverage the homeostasis maintenance of the neovascular and lymphatic network [57].

### Redox homeostasis

Oxidative stress mentioned above is an important link to mediate inflammatory response, which leads to vascular ECs damage through direct cytotoxic effects. The balance between reactive oxygen species (ROS) accumulation and nitric oxide (NO) consumption in hyperglycemia ultimately contributes to normal vascular endothelial function [58]. On one hand, the overproduction of ROS can be a central mediator of injury to cell constituents, including lipids, nucleic acids, and proteins [59]. The accumulated ROS subsequently induces signaling cascade of MAPK and activate NFκB, which results in expression of pro-inflammatory cytokines, chemokines and receptors [60, 61]. Lipids peroxidation, the denaturation of nuclear or mitochondrial DNA, as well as inactivated proteins after oxidative modification, together bring about substantial changes in membrane permeability and elasticity [59, 62]. On the other hand, NO (also called an endothelium-derived relaxing factor), is one of the most important vasodilator factors. It continuously maintains the dynamic balance of vascular endothelium by activating ERK1/2 and p44/p42 mitogen-activated protein kinases and causing tyrosine phosphorylation of focal adhesion kinase (FAK) [63]. Except for antihypertensive and antiplatelet effects, NO also inhibits LDL oxidation and leukocyte adhesion, thereby reducing the risk of endothelial thrombosis in patients with arteriosclerosis or diabetic CHD [64]. NO is synthesized from L-arginine under the catalysis of eNOS in vascular ECs [65]. However, some studies have shown that eNOS is mainly expressed in large vascular ECs, rarely in arterioles, and not in capillary ECs [30, 66]. Based on this structural expression, eNOS may not be involved in diabetic microangiopathy, but it is not clear at present.

### Metabolic homeostasis

Mitochondria are best known for cellular energy conversion and metabolic homeostasis. The balance of mitochondrial fusion and division, as well as normal autophagy, ensure the functional retention of mitochondria and timely clearance of damaged mitochondria, forming a highly dynamic regulatory mechanism against injury from DVCs [67, 68]. The OPA1 gene mediates mitochondrial fusion to protect ECs viability and reduce oxidative stress [68]. Normal release of ROS and stabilization of mitochondrial membrane potential reduce cell damage and aging, further preventing the vicious circle of mitochondrial damage caused by excessive peroxide in DVCs [67]. As for autophagy, VEGF equalizes autophagic flux through an AMPK/mTOR-dependent mechanism to prevent DVCs progression [69, 70].

Vascular ECs may also maintain their metabolic homeostasis by dynamically regulating ionic metabolite exchange. Iron homeostasis maintenance act as a vascular protector by reducing lipid peroxidation and excessive iron death [71, 72]. Zinc ions are required for eNOS dimerization and subsequent production of NO, while NO leads to the rapid mobilization of endothelial Zinc storage to mediate vascular cell protection and vasodilation [73]. Besides, other regulators Na^+^/H^+^ exchanger regulator-2 (NHERF-2) and second messenger Ca^2+^ also exist in the regulative process of endothelial homeostasis [74, 75].

## Common mechanisms of DVCs

### Effects of metabolic disorders on ECs

#### Glucose metabolism disorder

Glucose metabolism disorder acts as the initiating step and important risk factor of DVCs [76]. The continuous high glucose status and accumulation of intermediate metabolites increase mitochondrial membrane potential and ROS production. Four classical pathways (hyperactivated polyol pathway and hexosamine pathway replacing normal glucose metabolism pathway, PKC activation, accelerated production and accumulation of advanced glycation end products (AGEs), reviewed by Ighodaro [77]) exacerbate the damage to vascular endothelial function [78]. Among them, the most important AGEs are produced by non-enzymatic glycosylation reaction of proteins, lipids, DNA, and other substances in the human body with reducing sugars [79]. Excessive AGEs bind to their receptors (RAGE) in ECs. On one hand, TLR-4 heterodimerization activates the NF-κB pathway to upregulate the expression of adhesion molecules. Along with inflammatory cells and cytokines migrating and infiltrating local tissues, oxidative stress is prone to increase the permeability of blood vessel wall [80]. On the other hand, AGEs-RAGE leads to VEGF overexpression and abnormal hyperplasia of neovascularization [79, 81]. Overlapped multi-effects not only directly lead to damage of vascular ECs (such as peroxidation of biofilm lipids, destruction of cell structure and increased apoptosis), but also accelerates oxidative stress to prompt further damage to ECs [82].

#### Lipid metabolism disorders

Obesity-induced insulin resistance (IR) is reported as the common soil and intermediate connecting mechanism for glucolipid metabolism disorders [83]. Firstly, IR inhibits eNOS activity to reduce NO concentration through the PI3KA-MAPK pathway, which severely impairs endothelium-dependent vasodilation and increases its permeability [84]. In addition, IR stimulates the proliferation of VSMCs in turn and the excessive release of free fatty acid (FFA) in adipose tissue to aggravate oxidative stress and PKC activation, eventually forming a vicious circle [85]. Suppressed endothelial fatty acid oxidation (FAO) increases intracellular calcium oscillation. Those negative effects reduce NADPH levels and increase the ratio of NADP^+^/NADPH, resulting in vascular leakage, weakened endothelial antioxidant stress capacity and aggravation of endothelial activation caused by lipopolysaccharide (LPS) [86, 87]. Under the stimulation of hyperinsulinemia, the synthesis of lipoproteins in the liver increases. AGEs participate in modifying lipoproteins as accelerators of lipid metabolism disorders. Ultimately, AGEs increase TG-rich lipoproteins and LDL and decrease HDL [88].

The binding affinity between sdLDL and LDL receptors decreases, which hinders sdLDL clearance pathway [89]. Moreover, due to its small particles, it is easy to adhere to the subendothelial layer and combine with proteoglycan. Combined lipoproteins are more prone to oxidative modification. Stimulated macrophages in circulation and tissues phagocytize the modified LDL and turn them into foam cells, which eventually accelerates the development of DVCs under the pressure of immuno-inflammation [86, 89, 90].

#### Gut microbiome metabolites

The metabolites of intestinal flora, such as short-chain fatty acids (SCFA), bile acids (BA), branched-chain amino acids (BCAA), methylamines and gaseous transmitters affect host metabolism [91]. Intestinal flora disorders may help inhibit the reverse cholesterol transport mechanism (RCT). Excess production of LPS and foam cells then inhibits cholesterol efflux [92]. Other products indoxyl sulfate and trimethylamine-N-oxide (TMAO), lead to the deterioration of DVCs [93]. Indoxyl sulfate is produced in the liver from the metabolism of indole. Indole, known as a protein-imported uremic toxin, is converted from dietary tryptophan by bacterial tryptase in the colon. TMAO is the ramification of trimethylamine derived from the intestinal microbiota oxidated by flavin-containing monooxy geniuses and acts as the promoter of AS [92, 93].

#### Inflammatory metabolite-arachidonic acid derivatives

Arachidonic acid can be metabolized by three pathways: COX, LOX, and CYP4. The leukotriene, thromboxanes TXA2 and TXB2, and EET produced during arachidonic acid metabolism are involved in vascular-related diseases [94]. In the pro-inflammatory environment, PI3K/AKT signal pathway directly regulates the activity of NF-κB. Subsequently, NF-κB upregulates the inducible expression of genes including cyclooxygenase-2 (COX-2) and promotes caspase-3 activity that triggers apoptosis of ECs. COX-2 catalyzes arachidonic acid and produces numerous prostaglandins (PGs) [95]. PGI2 and TXA2 are different kinds of PGs produced by vascular ECs and platelets, respectively. PGI2 induces vasodilation and inhibits platelet aggregation, while TXA2 induces vasoconstriction. The imbalance between them is a key factor to disrupt endothelial homeostasis [96, 97]. Activation of thromboxane/endoperoxide receptors can also aggravate endothelial dysfunction [95]. Moreover, COX-2 activity interacts with oxidative stress. COX2 stimulates the production of non-phagocytic cell NADPH oxidase 2 (NOX2), and activates COX2 reversely, resulting in a continuous vicious cycle of endothelial dysfunction [98].

### Effect of immuno-inflammatory interaction on ECs

**Fig. 2:**
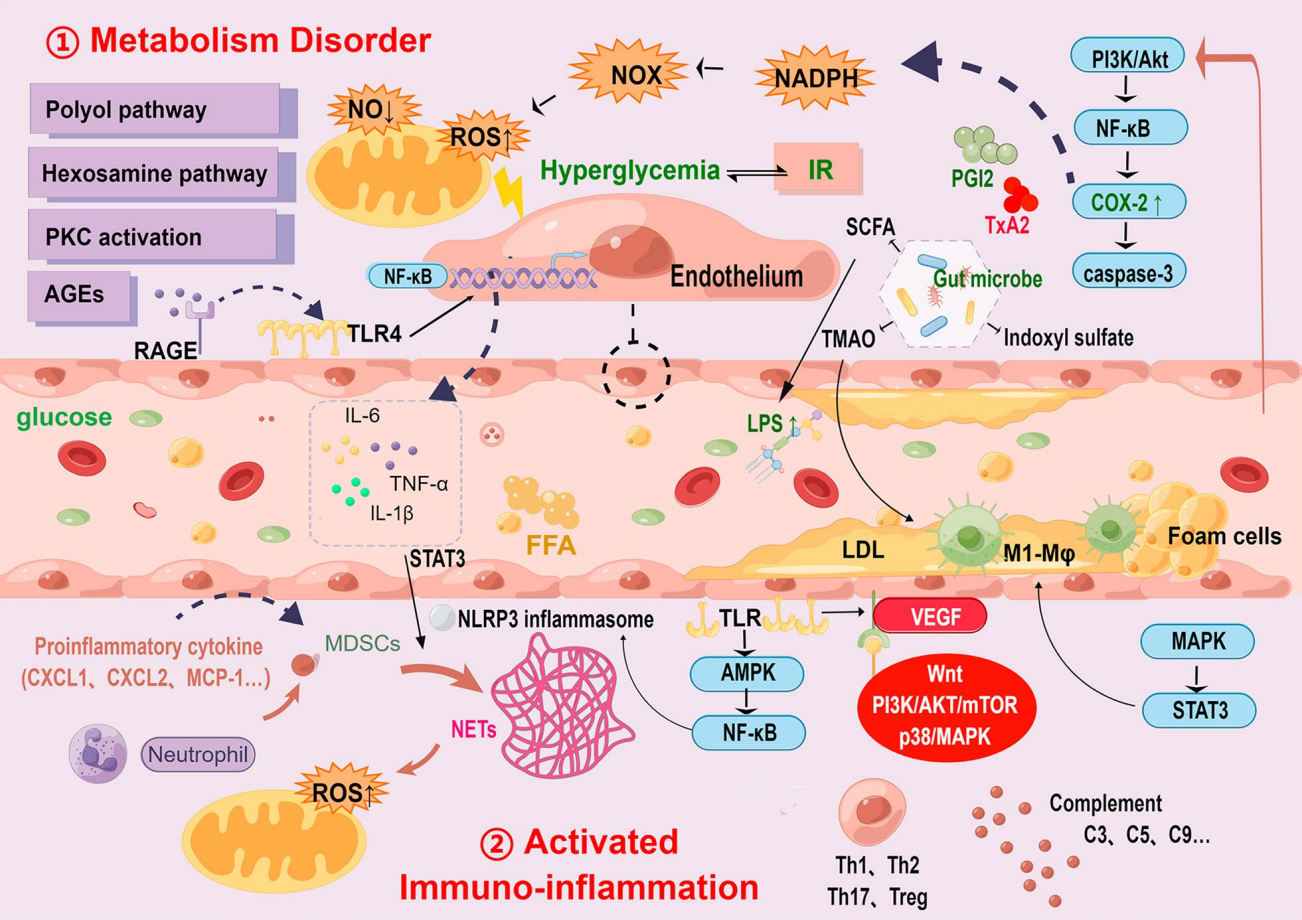
The interaction mechanism of metabolic disorders, immuno-inflammation and endothelial dysfunction in DVCs. The metabolic disorder process, characterized by hyperglycemia and IR, involves glucose, lipid, and gut microbe metabolism. The immuno-inflammation process is activated by immunocytes, proinflammatory cytokines, and NETs. Then induced inflammation and redox injury cause damage to ECs and vessels. AGEs: advanced glycation end products; CXCL: chemokine (C-X-C motif) ligand; FFA: free fatty acid; IR: insulin resistance; LPS: lipopolysaccharide; Mφ: macrophages; MCP-1: monocyte chemoattractant protein 1; MDSCs: marrow-derived myeloid cells; NETs: neutrophil extracellular traps; NO: nitric oxide; NOX: NADPH oxidase; PGI-2: prostaglandin I-2; PKC: protein kinase C; RAGE: receptor of AGEs; SCFA: short-chain fatty acids; Th: T helper; TLR: Toll-like receptors; TMAO: trimethylamine-N-oxide; Treg: T regulatory cells; TxA2: thromboxane A2; VEGF: vascular endothelial growth factor

Under the regulation of various cells and their secreted cytokines, TLR2/4-NF-κB, p38/MAPK, IL-6/STAT3 and other key transcriptional regulatory pathways get involved to constitute the immuno-inflammation interaction mechanism in DVCs (Fig. 2). Innate immunity is enhanced in patients with DVCs, yet adaptive immunity is relatively less studied.

#### Innate immune activation

##### Monocyte-macrophage system

As an important component of intrinsic immunity, the monocyte-macrophage system plays a key role in the chronic low-level inflammatory process [99]. Under the stimulus of metabolic disorders, Mφ polarized from anti-inflammatory M2-like macrophages (CD68^+^/Arg-1^+^) to pro-inflammatory M1-like macrophages (CD68^+^iNOS^+^) [100, 101]. M1-like macrophages secrete numerous proinflammatory cytokines (such as TNF-a, IL-1β, IL-6), chemokines (MCP-1/CCL-2) and inducible nitric oxide synthase (iNOS) [102]. Those cells, cytokines, chemokines and essential substances in tissue form an inflammatory M1-like immune microenvironment, maintaining a long-term inflammatory stress state of ECs [102, 103]. In parallel, metabolic disorders products FFA and LPS bind to membrane protein receptors of ECs and monocytes, leading to increased secretion of vascular cell adhesion molecules (intercellular adhesion molecule 1 (ICAM-1), etc.) and E-selectin [104]. Under the regulation of the AMPK-STAT3 axis, chemokines MCP-1 and CCR2 recruit circulating monocytes and M1-like differentiated macrophages to adhere to ECs in the vessel wall, accelerating metabolic disorders [103–105]. Infiltrating monocyte-macrophages transfer from the ECs gap to the tubular endothelial layer by rolling [104, 105]. Excess oxidized LDL can be phagocytosed and ingested by M1-like macrophages and VSMCs, which are the ultimate source of foam cell forms and AS plaques [106, 107]. Macrophages produce excessive metalloproteinases, which can dissolve and even rupture plaques [106, 108].

##### Granulocytes, MDSCs and NETs

The granulocyte system mainly includes neutrophils, eosinophils, and basophils. As a component of innate immunity, the granulocyte system often participates in immuno-inflammation by releasing proinflammatory mediators and cytokines [109]. Neutrophils are the most abundant inflammation-related immune cells in circulation and play a wide role in the inflammatory process [110]. Mechanisms of neutrophils that respond to immuno-inflammation in ECs mainly include the production of ROS, the secretion of inflammatory cytokines and peroxidase MPO, as well as the formation of NETs [111, 112]. With the spontaneous migration of granulocytes to targeted organs, the expression of adhesion molecules CD11b and ICAM-1 are also up-regulated [113, 114].

A special granulocyte system called proangiogenic granulocytes (PAGs) is composed of 20% neutrophils and 80% eosinophils [115]. PAGs are measured with CD49d^+^Ly6G^+^ adhesion marker on their surface and rich in VEGFR1 and CXCR4 molecules [116]. PAGs support the angiogenesis of ECs in vitro but PAG level is inversely proportional to blood glucose level [115]. The eosinophil count in DKD always rises, along with wider glomerular basement membrane (GBM) and positively correlated count with male albumin excretion rate [117, 118]. Eosinophils are rarely studied in other DVCs, and basophils are even rarely reported.

A large number of polymorphonuclear marrow-derived myeloid cells (PMN-MDSCs) are generated by bone marrow and spleen [112]. Under high glucose and chronic inflammatory conditions, IL-6/STAT3 pathway is activated. After recruitment by inflammatory chemokines (CXCL1, CXCL2, CXCL5, and S100A8/A9 (Calprotectin)), and activation by proinflammatory (IL-6, VEGF, IFN-γ, IL-1β, HMGB1), PMN-MDSCs transmit to inflammatory ECs to triggers neutrophil extracellular traps (NETs) [112, 119]. Up-regulated PMN-MDSCs express the components p47phox and gp91phox of NOX2. NOX2 not only accelerates the inflammatory response by excessive ROS production but also promotes the occurrence of NETs [102, 120]. Moreover, activated NLRP3 inflammasome also promotes NETosis in CHD and atherosclerosis [121]. That is an important mechanism of diabetic microangiopathy, which may be used as a potential therapeutic target.

##### The important role of other innate immune cells

Numerous previous studies have focused on the role of mast cells (MCs) in AS and CHD. It has been found that MCs have involved in vascular plaque formation and inflammatory infiltration to atherosclerotic vessel walls through cell cytoplasmic vacuolization, extracellular extrusion of granules and nuclear sequestration [122]. MCs can secrete various mediators to activate other inflammatory cells (such as lymphocytes and foam cells) and affect the metabolism and circulation of HDL and LDL [123, 124]. MCs activated by endogenous factors (ET-1, component C5R, etc.) can release proteases through granule exocytosis to degrade inflammatory markers and limit the progress of the inflammatory process [124].

DCs help to capture, process, and present antigens to antigen-presenting cells (APC). APC stimulates the activation and proliferation of antigen-specific T and B cells to initiate adaptive immunity [125, 126]. Activated dendritic cells (DCs) promote the secretion of proinflammatory mediators, including antibacterial mediators and chemokines, to help recruit more immune cells to infiltrate to local inflammatory vascular endothelium [125, 127]. In addition, DCs regulates the differentiation of T cells into different subsets [126].

##### Complement system

The systemic complement system is activated, and deposited serum membrane attack complex (MAC) results in cell damage to targeted organs [128–130]. In addition to inducing epithelial-mesenchymal transition and inflammatory cell infiltration, complement C3 and C5 also participate in transforming growth factor β (TGF-β) mediated endothelial EndMT [130, 131]. Complement regulator CD59, a membrane glycoprotein, can inhibit the polymerization of C9 to prevent the formation of MAC. CD59 is more susceptible to glucose-dependent nonenzymatic glycation-inactivation in DM. Autoantibodies against glycosylated proteins initiate complement activation again via classical pathways [132].

#### Specific immunity activation

Diverse immunosuppressive cells (like Treg cells) and secretory cytokines mainly composed the vascular endothelial immunosuppressive microenvironment of patients with DVCs [133, 134]. AGEs may act as a new epitope bound by the mannose-binding lectin (MBL) pathway to motivate adaptive immunity [135]. The balance of the circulating T lymphocyte subset is dynamically affected by hyperglycemia, with significantly increased CD4^+^ T cells and reduced CD8^+^ T cells [136]. Infiltrating CD8^+^ T cells in targeted tissues exerts negative regulatory effects on angiogenesis and endothelial function [137]. Moreover, microparticles released by T cells regulate the expression of eNOS and caveolin-1, accelerating endothelial dysfunction by NO and prostacyclin pathways [138].

T helper type 1 (Th1), Th2, Th17, regulatory T (Treg) cells, and cytotoxic T cells participate in the occurrence and development of DVCs, but the related molecular mechanism of endothelial dysfunction in various organs is not equal [139]. B cells contribute to the production of antibodies and cytokines, whereas few studies focus on the interconnection between them and inflammation in DVCs [140].

#### Immuno-inflammation related cytokines

##### Proinflammatory cytokines

The majority of proinflammatory cytokines infiltrated into local tissues originate from polarized M1-like macrophages or leukocytes [141, 142]. Toll-like receptors (TLRs) recognize damage-associated molecular patterns (DAMPs) released during cell stress and injury [143]. Combined TLR 2/4 in monocytes increases with NF-κB translocation to the nucleus and proinflammatory genes (encoding proinflammatory factors, like TNF-α, IL-6, IL-1β, IL-18) transcription. These changes result in NLRP3 inflammasome overexpression and NLRP3 activation-associated endothelial dysfunction [129, 144]. NLRP3 inflammasome also activates caspase-1 to increase the speed and severity of inflammation [145]. In this regard, TLR2/4 is positively correlated with transcription factor NF- κB expression and the severity of inflammation [146].

As one of the earliest detectable cytokines, TNF-α promotes the generation of ROS to aggravate intimal injury. The amount of TNF-α goes up with disease progression to form a vicious cycle. Its continuous secretion leads to the release of ICAM-1, which controls recruited macrophage infiltration [147]. Additionally, TNF-α is a key signaling molecule of the AMPK/NF-κB /NLRP3 pathway. Activation of NLRP3 inflammasome can trigger IL-1β and proIL-18 secretion to promote further inflammatory processes and oxidative stress [148]. Analogously, IL-6 interacts with TNF-α to enhance oxidative stress and reduce eNOS phosphorylation [149]. IL-6 receptor family activates JAK/STAT signal pathway to cooperatively regulate B cells differentiation, plasma cell genesis, and acute phase response [150, 151].

##### Chemokines

Chemokine MCP-1 and ICAM-1 can attract monocytes, macrophages, T cells, and DCs to inflammatory sites [139, 152]. MCP-1 promotes monocytes and macrophages activation and also upregulates the expression of adhesion molecules and proinflammatory cytokines [153, 154]. In addition, CXCL1, CXCL2, CXCL5, and calprotectin (S100A8/A9, calprotectin) are involved in neutrophil recruitment [155].

##### Other cytokines

VEGF is by far the strongest specific angiogenic factor and is also a signal communication bridge between islet β cells and ECs [66]. Under the condition of metabolic disorders, VEGF overexpression, mediated by various proinflammatory mediators, leads to increased vascular permeability, disordered angiogenesis, increased adhesion molecules, as well as broken barrier protection and function repair of ECs [156]. Multiple mechanisms related to VEGF include Wnt, PI3K/Akt/mTOR/eNOS and p38/MAPK [66, 156, 157]. TGF-β mainly participates in the process of ECs-related organ fibrosis. In kidney, retina, and heart fibrosis, disorders of glucose and lipid metabolism mediate EndMT in ECs through PKC β/TGF-β/PAI-1, TGF-β/SMAD, and a part of lncRNA signal pathways [158–161]. High glucose stimulation triggers ROS production and activates TGF-β/SAMD3 pathway. Thus, caused proinflammatory cytokines and chemokines upregulate fibroblast-stromal cell proteins by stimulating the AGE-RAGE axis [162].

#### The important role of oxidative stress in immuno-inflammation

Oxidative stress is associated with IR, B cell dysfunction, and damage to cell membrane integrity. Induced apoptosis, microvascular damage, and barrier break, ultimately lead to the progression of vascular complications [163, 164].

ROS in ECs is mainly generated by NOX2, mitochondrial respiratory chain, and eNOS uncoupling [165]. The systemic chronic inflammatory state induced by the disorders of glucose and lipid metabolism stimulates the overproduction of ROS from different sources. Endothelial NO inhibits NF-κB activation and upregulation of VCAM-1, E-selectin, and ICAM-2, thereby inhibiting leukocyte adhesion to vascular endothelium [166]. Total eNOS levels decreases, however, endothelial NO production is impaired and consumed by oxidative inactivation [64]. Imbalanced NO and ROS contribute to a vicious circle of oxidative stress. Pathways and molecules involved in this process include the polyol pathway, MAPK, NF-KB, forkhead box O (FOXO), Keap1-Nrf2/ARE, Nrf2/HO signal pathway and so on [90, 167–169]. Oxidative stress disturbs endothelial signal transduction and enhances vascular endothelial permeability and leukocyte adhesion through these above mechanisms, which ultimately exacerbates vascular endothelial dysfunction [167].

### Interaction between metabolic disorder and immuno-inflammation

Increased levels of circulating inflammatory biomarkers in DM patients appear to predict the onset and progression of DVCs [170]. Long-term high glucose environment triggers innate immunity and homeostasis maintaining response once AGEs are recognized by pattern recognition receptors (PRRS). Induced abnormal function of mitochondria and endoplasmic reticulum promotes oxidative stress and endothelium structural damage. The process of oxidative stress is related to the elevated levels of proinflammatory cytokines and autoantibodies. AGEs/RAGE pathway activates downstream NF-κB signal pathway to stimulate blood vessels continuously.

Lipotoxicity leads to lipid metabolism products and visceral adipose tissue levels dynamic storage addition. These products induce M1 polarization of macrophages. LDL adhesion remains in the subendothelial layer of blood vessels and receives oxidative modification to oxidized low-density lipoprotein (ox-LDL), which can be engulfed by M1-like macrophages and transformed into foam cells. The expression of systemic inflammatory cytokines increases, to form a chronic low-level inflammatory state in the body, which runs through the process of DVCs.

## Downstream effector mechanisms of DKD, DR and DHD

**Fig. 3:**
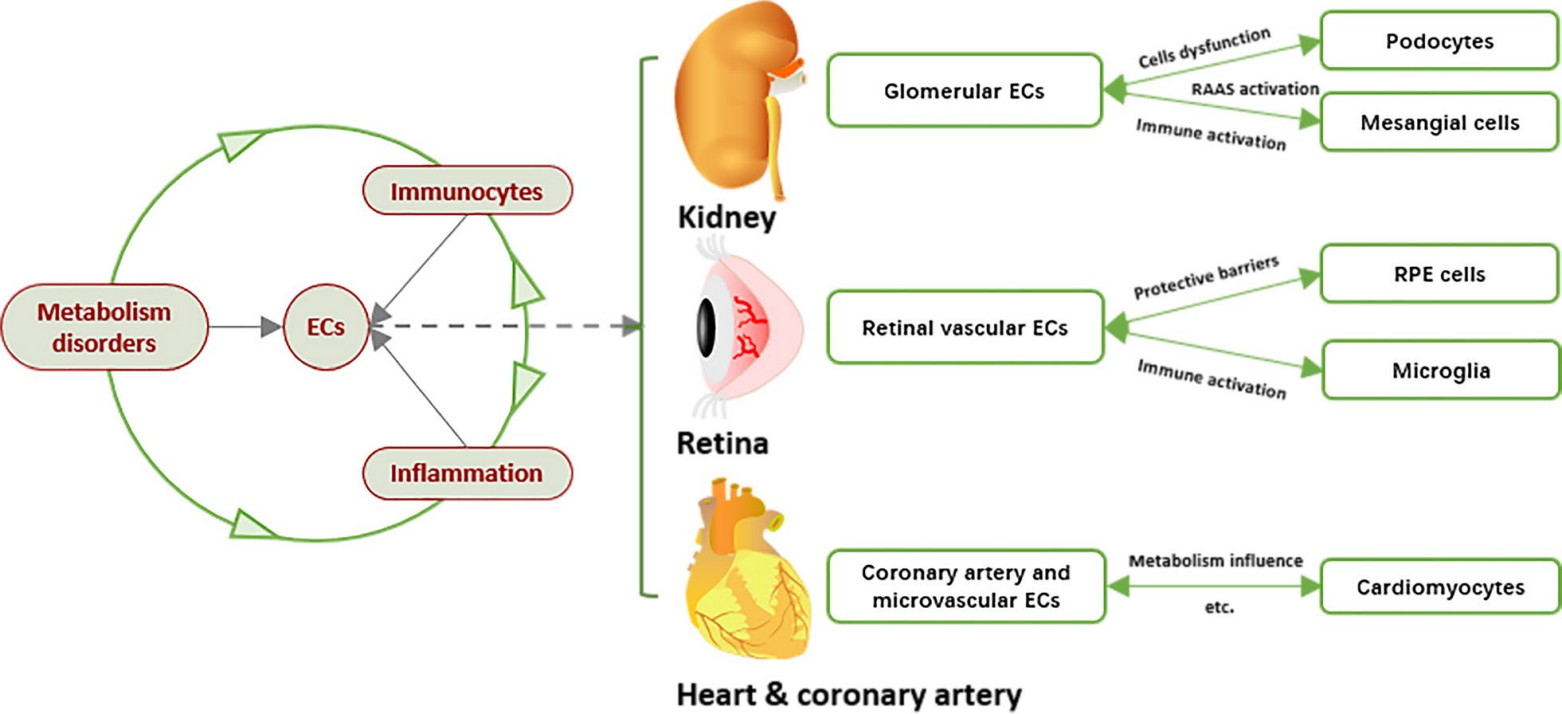
The inner linkage of endothelial dysfunction and different DVCs. The complex relationship of metabolic disorders, immuno-inflammation and endothelial dysfunction (left part) and different downstream effector mechanisms of DKD, DR and DHD (right part) are shown. ECs: endothelial cells; RAAS: renin-angiotensin aldosterone system; RPE: retinal pigment epithelial

Different targeted organs consist of different cells. Inevitably, the main functional cells of kidney, retina, heart and coronary artery seem to be affected by endothelial dysfunction (Fig. 3).

### Glomerular ECs, podocytes, mesangial cells and DKD

As the most common diabetic microangiopathy, DKD developed with the cellular structure destruction and dysfunction of glomerular ECs, podocytes, and mesangial cells. VEGF secreted by ECs or podocytes binds to its receptors VEGFR, regulating the normal vascular permeability and angiogenic function [171]. Once ECs are damaged, the function of renal mesangial cells will be seriously affected along with plenty of podocytes lost. Besides, activated local renin-angiotensin aldosterone system (RAAS) results in the consequences of glomerular hyperperfusion, high pressure and high filtration. Other mechanisms include immune activation related to immune complex deposition, inflammatory response caused by the chemokines serum amyloid A (SAA) and regulated upon activation of normal T-cell expressed and secreted (RANTES) [172, 173], as well as the profibrotic cascade with multiple cytokines involved [174, 175].

### Retinal vascular ECs, RPE cells, microglia and DR

The high metabolic demand and limited vascular supply to retinal cells make them extremely sensitive to metabolic disorders. The disorder of vascular structure is more significant in DR for the influence of VEGF overexpression [8]. Thrombus and haemorrhage coexist after the formation of microangioma [176]. Even fibrovascular membrane hyperplasia or traction retinal detachment occurs in the proliferative stage, accompanied by retinal neurodegeneration [177]. In fact, the retina is an immune privilege tissue. The blood-retinal barrier (BRB) is formed by a tight junction of retinal vascular ECs (inner BRB) and retinal pigment epithelial (RPE) cells (outer BRB) [188]. Unless BRB structural disruption in severe DR, circulating cells and molecules cannot freely enter the retinal parenchyma, much less specific autoantibodies or T cell infiltration [178]. Additionally, there is no lymphatic system in the retina so that it is difficult to recognize DAMPs.

Retinal microglia are known as the most important local immune cells in DR. Others include perivascular macrophages, persistent transparent cells and DCs. Hyperglycemia inhibits the activation of myeloid cells (microglia/macrophages) and T cells, but it can induce Tregs cells formation through CD200-CD200R or CX3CLL-CX3CRL pathway [179, 180]. RPE cells induce apoptosis of infiltrating immune cells through FAS ligand and TRAIL pathway. RPE cells also inhibit complement activation with the help of CD55, CD46 and CD59. Recently, it has been found that CD11b^+^ monocytes in the peripheral blood of DM mice were more active and expressed higher levels of the chemokine receptor CCR5 [181]. They preferentially stay in retinal microvessels and may be the risk factor for DR.

### Coronary artery and microvascular ECs, cardiomyocytes and DHD

Adverse outcomes of diabetic heart disease (DHD) include significant reduction of glucose supply and utilization, depletion of glucose transporter 4 (GLUT4) mediated PPARs pathway, pyruvate dehydrogenase activity inhibition by β-oxidated FFA, the burden of lipid metabolism in cardiomyocytes aggravated by ubiquitin ligase MG53, and cardiac systolic and diastolic dysfunction caused by myocardial lipotoxicity intermediates such as ceramide [182–185]. There are intimate relationships and intercellular dependence between ECs and cardiomyocytes. DM-related coronary heart disease is mainly associated with atherosclerosis caused by glucose and lipid metabolism disorders, whereas the targeted cells of diabetic cardiomyopathy are mainly cardiomyocytes. Cardiomyocytes are more sensitive to energy metabolism and ion changes, among which Ca^2+^ is the main ion causing myocardial contraction. Endoplasmic reticulum stress promotes the disorder of Ca^2+^ metabolism in the cardiomyocyte membrane and eventually causes cardiomyocyte death [186, 187].

## Discussion

Vascular disease is a leading cause of death and disability for DM patients worldwide [188]. The burden of DM and its complications are unmet public health issues. Secondary prevention of DM, which means preventing diabetic complications and decreasing risks of major organ injury, is immediately needed. If so, a large quantity of DM patients might have the opportunity to escape from those bad consequences. However, the absence of suitable drugs for the prevention of major vascular events after DM leaves clinicians with dilemmas. For example, ACEI/ARB drugs could not prevent disease progression in the kidneys but increase the incidence of cardiovascular events [189, 190]. Aspirin and clopidogrel for primary and secondary prevention of CHD are recommended, while few proofs of they improve microvascular complications [191, 192].

### Proofs of DVCs prevention

Potential preclinical interventions (Table 1) bring new light on realizing secondary prevention of DM. These potential medications were associated with a possible structural benefit in administration of different DVCs. With respect to diabetes-related CHD, combined secondary prevention practices of CHD (e.g., antiplatelet, lipid-lowering, blood pressure control and lifestyle intervention) and antidiabetic drugs without cardiovascular risk have been proposed [191]. The scope of the research on diabetes-related CHD gradually increases and present studies are carried out around therapies of novel glucose-lowering drugs, lipid regulators, antithrombotic drugs, and so on [193]. Eventually, these data should be confirmed with prospective, randomized, controlled clinical trials.

**Table 1:** Potential preventive intervention for DVCs from experimental data

Disease	Potential preventive intervention from experimental data	References
DKD	Natural products (quercetin, fisetin, triptolide, sappanone A, ginkgo biloba leaf extract, lespedeza bicolor, silymarin and milk thistle extract) Herbal prescription (TangShen WeiNing formula, Ayurvedic formula) Metabolites (butyrate, polysulfides, thiamine) SGLT2 inhibitors, calcium dobesilate, spironolactone, irbesartan	[194–207]
DR	Natural products (pterostilbene, puerarin Herbal prescription (Yiqi Tongluo Fang, Ayurvedic formula) Montelukast	[208–212]
DC	Metabolites (zinc supplementation) trimetazidine, telmisartan and thiorphan combination treatment, SGLT2 inhibitors, CaMKII inhibitors, FGF1^ΔHBS^ treatment	[213–218]

*DKD* diabetic kidney disease; *DR* diabetic retinopathy; *DCM* diabetic cardiomyopathy; *DVCs* diabetic vascular complications

A retrospective summary of registered clinical trials (WHO international clinical trials registry platform, https://trialsearch.who.int/) on the prevention of DVCs (Table 2) demonstrates that evidence of both safety and effectiveness is still absent. PRIORITY is a multicentre, prospective, observational randomised controlled trial initiated by Steno Diabetes Center [219]. This study illustrated that spironolactone could not prevent progression to microalbuminuria and diabetic kidney disease, which is contrary to what the previous experiment showed [195, 219]. Another exciting trial PRECIDENTD is currently in progress and is due to be completed in 2028. Up-to-now guidelines recommend the initiation of SGLT2 inhibitors or GLP1 receptor agonists with proven cardiovascular benefits in adult patients with T2DM [220]. PRECIDENTD trial will innovatively compare two drugs to prevent cardiovascular and diabetic kidney disease.

**Table 2:** Registered clinical trials on the prevention of diabetic vascular complications

Main ID	Country	Invention	Prevention	Status
PRECIDENTD, NCT05390892	United States	GLP-1 receptor agonist and/or SGLT2 inhibitor	Cardiovascular and diabetic kidney disease	Ongoing
EUCTR2019-004772-19-DK	Denmark	Hjertemagnyl	Diabetic kidney disease	Ongoing
CTRI/2015/01/005366	India	Ayurvedic formulation	Diabetic vascular complications	Ongoing
PRIORITY, NCT02040441/UCTR2012-000452-34-GB/DRKS00008801	Multi-European countries	Spironolactone	Diabetic kidney disease	Completed
NCT01725412	Canada	Thiamine	Diabetic kidney disease	Unknown
JPRN-UMIN000007718	Japan	Antiplatelets (cilistazol)	Diabetic kidney disease	Unpublished
JPRN-UMIN000002718	Japan	Olmesartan	Diabetic kidney disease	Unknown

### Possibilities and challenges of parallel targets in DVCs

*Treatment of metabolic disorders* is based on lifestyle intervention and patient education [221]. By controlling blood glucose, blood pressure, blood lipids and other risk factors, it is expected that DM patients keep a healthy lifestyle and get effective follow-up care strategies. Loss of body weight and drugs assist with ideal metabolic control to protect the function of vital targeted organs [222]. SGLT2 inhibitors are high-profile glycaemic control drugs and associated with reductions in body mass and blood pressure, as well as with both kidney and cardiovascular protective effects [223]. Remarkably, metabolism regulation makes a meaningful impact on both primary and secondary prevention of DM.

*Treatment on immunity and inflammation mechanisms* includes extracts from Traditional Chinese Medicine (TCM, like rhein, hirudin, polysaccharides), SGLT2 inhibitors, ACEI/ARB agents and so on. TCM therapy has unique advantages for DVCs prevention, which still deserves further study [224]. Rhein, derived from the roots of *Rheum L.,* protects islets β cells by inhibiting abnormal activation of the hexosamine biosynthesis pathway and reverse IR. Besides, Rhein antagonizes TGF-β and protects ECs function [225]. Hirudin, an acidic polypeptide secreted by the salivary glands of *Hirudo medicinalis*, inhibits HIF1α/VEGF and p38 MAPK/NF-κB pathways and activates NRF-2/HO-1 pathways to prevent DN and other vascular complications [226]. Polysaccharides from different Chinese herbs have significant anti-diabetic and anti-DVCs effects through various mechanisms with almost no side effects [227]. Similarly, SGLT2 inhibitors also modify inflammatory responses by various underlying mechanisms (e.g., oxidative stress, RAAS activation, and immune system function) [223]. Moreover, clinical investigators have been always trying to use ACEI/ARB drugs for secondary prevention of DM, but results are still controversial. ACEI/ARB drugs reduce the formation of angiotensin II or prevent angiotensin II from binding to angiotensin receptors to inhibit RAAS system [228]. They also help relieve local inflammation on the vessel wall to reduce urine protein and regulate blood pressure for patients with DVCs, yet losartan probably increases incidence of macroalbuminuria [229, 230]. Whether other ACEI/ARB use should be recommended for secondary prevention of DM need support from further research data.

*Treatment of the endothelial injury* ET-1 receptor inhibitors (avosentan, atrasentan, etc.) have been used to lower blood pressure and urine protein, but increase the risk of edema and heart failure [231–233]. Moreover, the effects of ET-1 receptor inhibitors for DKD and DHD lack clinical trial validation. Vasodilators can prevent DVCs and their progression by improving the microcirculation of the kidney, retina and myocardium [234]. Pancreatic kallidinogenase (PK), a vital vasodilator, increases capillary blood and tissue perfusion flow by dilating small pulsations and regulating blood rheology. It is reported that PK is beneficial to control the process of DKD, DR and DHD [235–237]. The mechanisms of PK may involve the protection of ECs, the influence of angiogenesis and permeability, as well as the reduction of tissue fibrosis [237–240].

### Opportunities for new therapeutics and secondary prevention

In the future, an improved understanding of the integration and regulation of the crosstalk network between metabolic disorder, immuno-inflammation and endothelial dysfunction may provide novel and effective therapeutic targets for DVCs prevention. If effective interventions can be taken on those common mechanisms, multi-disciplinary comprehensive therapy of DM-related vascular disease may provide new ideas for further improved clinical efficacy. For now, though, this is still up for debate. Therefore, the combinatorial drug treatment on the strong interaction network or natural products like TCM prescriptions characterized by multi-component, multi-target, multi-path comprehensive prevention and treatment of diseases may be a preferable alternative.

## Data Availability

Not applicable.

## Abbreviations

ACEI: Angiotensin-converting enzyme inhibitor
AGEs: Advanced glycation end products
AJ: Adhesive junction
AMPK: Adenosine 5′-monophosphate (AMP)-activated protein kinase
ARB: Angiotensin receptor blocker
AS: Atherosclerosis
BA: Bile acids
BCAA: Branched-chain amino acids
CACs: Circulating angiogenic cells
CHD: Coronary heart disease
COX-2: Cyclooxygenase-2
DCM: Diabetic cardiomyopathy
DCs: Dendritic cells
DHD: Diabetic heart disease
DKD: Diabetic kidney disease
DM: Diabetes mellitus
DR: Diabetic retinopathy
DVCs: Diabetic vascular complications
ECs: Endothelial cells
EGC: Endothelial glycocalyx
eNOS: Endothelial nitric oxide synthase
ERK: Extracellular regulated protein kinases
FAK: Focal adhesion kinase
FFA: Free fatty acid
FAO: Fatty acid oxidation
GJ: Gap junction
GLUT-4: Glucose transporter 4
ICAM-1: Intercellular cell adhesion molecule 1
IL: Interleukin
iNOS: Inducible nitric oxide synthase
IR: Insulin resistance
LDL: Low-density lipoprotein
LPS: Lipopolysaccharide
MAPK: Mitogen-activated protein kinase
MCP-1: Monocyte chemoattractant protein 1
MCs: Mast cells
MEF-2: Myocyte Enhancer Factor 2
mTOR: Mechanistic target of rapamycin
mtorc1: MTOR complex 1
NO: Nitric oxide
NOX2: NADPH oxidase 2
NETs: Neutrophil extracellular traps
NRP-1: Neuropilin-1
OS: Oscillatory shear
PAGs: Proangiogenic granulocytes
PGs: Prostaglandins
PKC: Protein kinase C
PMN-MDSCs: Polymorphonuclear marrow-derived myeloid cells
PS: Pulsatile shear
RAAS: Renin angiotensin aldosterone system
RhoA: Ras Homolog Family Member A
ROS: Reactive oxygen species
S1P: Sphingosine 1-phosphate
S1PR1: Sphingosine 1-phosphate receptor 1
SCFA: Short-chain fatty acids
SGLT2: Sodium-dependent glucose transporters 2
STAT3: Signal transducer and activator of transcription 3
TAP: Yes-Associated Protein
TGF-β: Transforming growth factor β
TJ: Tight junction
TMAO: Trimethylamine-N-oxide
TNF-α: Tumor necrosis factor α
VEGF: Vascular endothelial growth factor
VEGFR2: VEGF receptor 2
VSMCs: Vascular smooth muscle cells
